# Clinical and Histopathological Correlation of Quantitative HBsAg Levels in Chronic Hepatitis B

**DOI:** 10.1155/ijh/9096871

**Published:** 2025-07-28

**Authors:** Turgay Yılmaz, Erdoğan Özdemir, Deccane Düzenci, İbrahim Halil Bahçecioğlu

**Affiliations:** ^1^Department of Internal Medicine, Fethi Sekin City Hospital, Elazig, Turkey; ^2^Department of Intensive Care Unit, Fethi Sekin City Hospital, Elazig, Turkey; ^3^Department of Gastroenterology, Firat University Hospital, Elazig, Turkey

**Keywords:** chronic hepatitis B, histopathology, quantitative HBsAg

## Abstract

**Objective:** This study is aimed at comparing different clinical forms of chronic hepatitis B (CHB) infection (HBeAg-negative chronic HBV infection and HBeAg-positive and HBeAg-negative CHB patients) and evaluate their demographic, laboratory, virological, and histopathological characteristics, as well as investigate the relationship between quantitative HBsAg (qHBsAg) levels and these parameters.

**Materials and Methods:** This prospective study included a total of 307 patients, comprising 142 HBeAg-negative chronic HBV infection and 165 CHB patients (39 HBeAg-positive and 126 HBeAg-negative). Patient data, including age, sex, ALT, AST, GGT, ALP, total bilirubin, HBV DNA, and qHBsAg levels, were recorded. Additionally, liver biopsy was performed in 111 cases (31 HBeAg-positive and 80 HBeAg-negative), and histological activity index (HAI) and fibrosis staging (ISHAK score) were evaluated.

**Results:** No significant differences were observed between HBeAg-negative chronic HBV infection and CHB patients in age and sex distribution. In the CHB group, ALT and HBV DNA levels were significantly higher (*p* = 0.014 and *p* = 0.025, respectively). Among CHB patients, HBeAg-positive patients had significantly lower qHBsAg levels than HBeAg-negative patients (1805 vs. 4028 IU/mL, *p* < 0.001). Histopathological evaluations showed no significant association between qHBsAg levels and fibrosis severity (ISHAK score > 2) or necroinflammatory activity (HAI > 6). ROC analysis confirmed the limited diagnostic value of qHBsAg for advanced fibrosis (AUC 0.511, 95% CI 0.454–0.569). In HBeAg-positive patients, a weak negative correlation was found between qHBsAg and HBV DNA levels (*r* = –0.388, *p* = 0.015).

**Discussion:** Our study demonstrated variability in laboratory findings across different forms of CHB. Notably, HBeAg-positive patients exhibited high HBV DNA levels alongside low qHBsAg levels. The limited efficacy of qHBsAg as a fibrosis marker suggests caution in its clinical use. These findings underscore the importance of considering multiple parameters in assessing liver damage.

## 1. Introduction

Hepatitis B virus (HBV) is a DNA virus that is prevalent worldwide and can lead to liver diseases. Approximately 400 million people globally are infected with HBV, with a significant proportion carrying chronic infection [1].

Chronic HBV infection can cause progressive liver disease. The diagnosis and staging rely on key parameters such as the presence of serum HBsAg and HBV DNA, liver function tests, and liver histopathology. The course of the disease may vary, including phases such as HBeAg-positive chronic HBV infection, HBeAg-positive chronic hepatitis B (CHB), HBeAg-negative chronic HBV infection, or HBeAg-negative CHB. For a diagnosis of CHB, the following criteria must be met: HBsAg positivity persisting for more than 6 months, detectable HBV DNA levels, elevated transaminases, and histopathological evidence of moderate to severe necroinflammatory activity and/or fibrosis [2].

Treatment decisions and response assessments are based on liver function tests, HBV DNA levels, and fibrosis scores. The primary goals of treatment are to suppress viral replication, prevent liver damage, and reduce the risk of complications [3].

Liver fibrosis is one of the most critical complications of CHB and plays a key role in determining disease prognosis. Although liver biopsy remains the gold standard for fibrosis assessment, its invasive nature and associated complications have increased the demand for non-invasive alternatives. In this context, the potential use of quantitative HBsAg (qHBsAg) levels as a fibrosis marker is being investigated [4].

This study compares the demographic, laboratory, and virological characteristics of HBeAg-negative chronic HBV infection, HBeAg-positive CHB, and HBeAg-negative CHB patients. Additionally, the relationship between serum HBsAg levels, HBV DNA levels, and liver histopathology was examined to evaluate whether HBsAg quantification can serve as a predictive marker for disease activity and stage.

## 2. Materials and Methods

A total of 307 treatment-naive, HBV-infected volunteer patients aged ≥ 18 years who presented to the Gastroenterology outpatient clinic were included in the study. Ethical approval was obtained from the Ethics Committee of Firat University Faculty of Medicine (Approval No: 4561).

Patients were evaluated for HBsAg, HBeAg, anti-HBe, and HBV DNA levels and categorized into the following groups:

• HBeAg-negative chronic HBV infection: HBsAg (+), HBeAg (−), anti-HBe (+), and HBV DNA < 2000 IU/mL.
• CHB patients: HBsAg (+) and HBV DNA > 2000 IU/mL, further subdivided into:
  o. HBeAg-positive CHB: HBsAg (+), HBeAg (+), and HBV DNA > 2000 IU/mL.
  o. HBeAg-negative CHB: HBsAg (+), HBeAg (−), anti-HBe (+), and HBV DNA > 2000 IU/mL

The study population consisted of 142 HBeAg-negative chronic HBV infections and 165 CHB patients (39 HBeAg-positive and 126 HBeAg-negative). Liver biopsy was performed in 31 HBeAg-positive and 80 HBeAg-negative CHB patients, as some patients declined the procedure.

### 2.1. Exclusion Criteria

• Coinfection with HIV, hepatitis D virus (HDV), or HCV
• Decompensated liver disease
• Serum creatinine > 1.5 mg/dL
• Age < 18 years
• Pregnancy

### 2.2. Measurement of Biochemical Parameters

Biochemical parameters (fasting blood glucose, total protein, urea, creatinine, albumin, total bilirubin, LDH, ALT, AST, ALP, and GGT) were measured using a SIEMENS ADVIA 2400 autoanalyzer and SIEMENS commercial kits.

### 2.3. Measurement of Serological and Virological Parameters

Serological markers (HBsAg, HBeAg, anti-HBe, anti-HBc IgG, anti-HBs, anti-HCV, and anti-delta) were assessed using macro-ELISA on the ABBOTT ARCHITECT i1000SR system. HBV DNA levels were quantified by real-time PCR using the QIAGEN SYSTEM (Germany) and QIAsymphony kits.

### 2.4. Liver Biopsy and Histopathological Evaluation

Liver biopsy specimens were evaluated under light microscopy at the Pathology Laboratory of Firat University Faculty of Medicine using the Modified Knodell (Ishak) scoring system.

### 2.5. Statistical Analysis

Statistical analyses were performed using SPSS. Data was presented as numbers, percentages, means, and standard deviations. An independent samples *t*-test was used for normally distributed continuous variables. Pearson's correlation analysis assessed relationships between variables. The Tukey HSD test was applied for multiple group comparisons.

ROC (receiver operating characteristic) analysis was conducted to evaluate the predictive performance of HBsAg levels in detecting advanced fibrosis (Ishak score > 2, HAI > 6). The area under the ROC curve (AUC) and optimal cut-off values were calculated.

Statistical significance was set at *p* < 0.05.

## 3. Results

A total of 307 HBV-infected cases were analyzed. Among them, 142 were HBeAg-negative chronic HBV infections and 165 were CHB patients. Of the CHB patients, 126 were HBeAg-negative and 39 were HBeAg-positive. There were no significant differences between HBeAg-negative chronic HBV infections and CHB patients in terms of AST, ALP, GGT, total protein, albumin, total bilirubin, direct bilirubin, urea, creatinine, and HBsAg levels. However, ALT and HBV DNA levels were significantly higher in the CHB group (*p* = 0.014 and *p* = 0.025, respectively) (Table 1).

When comparing HBeAg-positive and HBeAg-negative CHB patients, HBeAg-positive patients were found to be younger and had significantly higher levels of AST, ALT, ALP, total bilirubin, direct bilirubin, and HBV DNA. Conversely, HBsAg levels were significantly lower in HBeAg-positive patients compared to HBeAg-negative patients (*p* < 0.001) (Table 2).

When comparing HBeAg-negative chronic HBV infection and HBeAg-positive CHB patients, ALT, total bilirubin, direct bilirubin, and HBV DNA levels were significantly higher in HBeAg-positive CHB patients, while HBsAg levels were significantly lower (*p* < 0.001). Age was higher in HBeAg-negative chronic HBV infection (Table 3).

In comparison between HBeAg-negative chronic HBV infection and HBeAg-negative CHB patients, there were no significant differences in age, biochemical parameters, viral load, or HBsAg levels (Table 4).

A total of 111 patients who underwent histological evaluation (31 HBeAg-positive and 80 HBeAg-negative) were categorized according to two distinct scoring systems based on their histopathological findings. According to the Histological Activity Index (HAI), scores between 0 and 6 were classified as Group 1 (mild activity), and scores between 7 and 18 were classified as Group 2 (moderate/severe activity). Similarly, based on the ISHAK fibrosis scoring system, scores between 0 and 2 were categorized as Group 1 (mild fibrosis), and scores between 3 and 6 as Group 2 (advanced fibrosis). This classification was applied separately for both HBeAg-positive and HBeAg-negative patients.

qHBsAg and HBV DNA levels were compared between the HAI and ISHAK score groups in both HBeAg-positive and HBeAg-negative patients. In the HBeAg-positive group, qHBsAg levels were significantly higher in the HAI-2 group compared to HAI-1 (*p* = 0.008), while the difference between ISHAK groups showed borderline significance (*p* = 0.079). HBV DNA levels did not show a significant difference across either scoring system. In the HBeAg-negative group, neither qHBsAg nor HBV DNA levels differed significantly between HAI groups (*p* > 0.05), whereas HBV DNA levels were significantly higher in the ISHAK-2 group compared to ISHAK-1 (*p* = 0.006) (Table 5).

**Figure 1:**
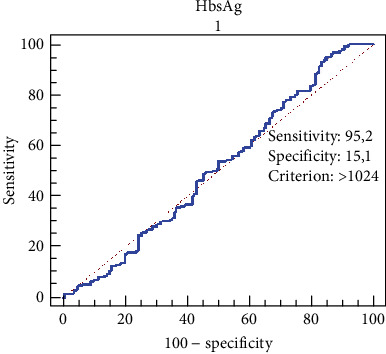
ROC curve of HBsAg levels for predicting advanced fibrosis. In the ROC curve analysis, the AUC for qHBsAg levels in predicting advanced fibrosis was calculated as 0.511 (95% CI: 0.454–0.569, SE: 0.0333), indicating no statistically significant diagnostic performance (*Z* = 0.341, *p* = 0.7332).

The predictive power of HBsAg levels for advanced fibrosis (ISHAK score > 2) was evaluated using ROC analysis. The performance of HBsAg in distinguishing advanced fibrosis was found to be low (AUC: 0.511; 95% confidence interval: 0.454–0.569). The cut-off value was determined as 1024 IU/mL. At this threshold, the sensitivity was calculated as 95.2%, while the specificity was 15.1% (Figure 1).

Additionally, our study investigated the relationship between patients' qHBsAg levels and HBV DNA levels. No correlation was observed between HBsAg and HBV DNA levels in HBeAg-negative chronic HBV infection and anti-HBe positive patients. However, a weak negative correlation was found between qHBsAg and HBV DNA levels in HBeAg-positive patients (*p* = 0.015; *r* = –0.388).

## 4. Discussion

Recently, numerous studies have focused on qHBsAg levels. These studies suggest that qHBsAg may be useful as a predictor of treatment response both before and during therapy [5]. Thompson et al. [6] reported that qHBsAg and quantitative HBeAg levels can serve as markers at the initiation and during the follow-up of therapy.

HBsAg is used not only for the diagnosis of hepatitis B infection but also for the evaluation of clinical and treatment outcomes. HBsAg clearance has been associated with better clinical outcomes. In CHB, the loss of HBsAg typically occurs in conjunction with clinical improvement, and the suppression of viral replication along with HBsAg or HBeAg plays a critical role in assessing treatment success and duration [7, 8].

This study is aimed at comparing different clinical forms of CHB infection (HBeAg-negative chronic HBV infection, HBeAg-positive, and HBeAg-negative CHB patients) and investigate their demographic, laboratory, and histopathological characteristics as well as the relationship with qHBsAg levels. The findings show similarities with existing literature while also presenting some unique results.

In our study, the mean age of all patients was 41.75 years, with 42.95 years for HBeAg-negative chronic HBV infection and 41.04 years for CHB patients, showing no statistically significant difference. In a study by Yuen et al. [9], the mean age was reported as 37.2 years in HBeAg-positive CHB patients and 45.2 years in HBeAg-negative CHB patients. In both groups, male sex was predominant.

In our cohort, the mean age of HBeAg-positive CHB patients was significantly lower than that of HBeAg-negative CHB patients and HBeAg-negative chronic HBV infection (*p* < 0.01). These age differences may reflect the fact that HBeAg positivity corresponds to the active phase of infection, whereas the older average age in HBeAg-negative patients and HBeAg-negative chronic HBV infection likely reflects the long-term natural course of CHB [10].

There was no significant difference in age, GGT, ALP, or total bilirubin levels between HBeAg-negative chronic HBV infection and CHB patients in our study. However, ALT and HBV DNA levels were significantly higher in CHB patients. The mean ALT level was 29.9 IU/L in HBeAg-negative chronic HBV infection and 74.17 IU/L in CHB patients, with this difference being statistically significant. In HBeAg-positive patients, the mean ALT level was 142.1 IU/L, which was significantly higher than in anti-HBe-positive patients and HBeAg-negative chronic HBV infection (*p* < 0.01). This finding supports that HBeAg-negative chronic HBV infection is characterized by low viral replication and minimal liver damage and highlights the elevated disease activity in HBeAg-positive patients [11]. In line with the literature, our study confirms that ALT and HBV DNA levels are markers of disease activity [12].

The finding that HBV DNA levels were higher in HBeAg-positive patients compared to HBeAg-negative ones is consistent with the literature and demonstrates more active viral replication in the HBeAg-positive group [13]. Interestingly, qHBsAg levels were found to be lower in HBeAg-positive patients in our study. This may suggest that viral protein production in HBeAg-positive patients is regulated by different mechanisms. Furthermore, the fact that necroinflammation and fibrosis severity did not differ significantly between HBeAg-positive and HBeAg-negative patients suggests that HBeAg status alone may not be a sufficient marker of disease progression [14].

Several studies have reported that HBsAg levels vary depending on the phase of disease and HBV genotype [15, 16]. In our study, the mean HBsAg levels were 3573.58 IU/mL in patients with HBeAg-negative chronic HBV infection. According to the EASL 2025 guideline, a qHBsAg level below 1000 IU/mL together with low HBV DNA is suggested to identify this infection phase with high diagnostic accuracy in Asian and European cohorts [2]. However, our results demonstrated considerably higher qHBsAg levels despite low HBV DNA and minimal histological activity. This discrepancy may reflect Genotype D predominance in our population, differences in individual immune response, or laboratory assay calibration variability. Therefore, these findings indicate that qHBsAg alone may not reliably define the low replicative phase in genotype D–dominant settings.

The mean qHBsAg levels were measured as 1805.17 IU/mL in HBeAg-positive CHB patients and 4028.61 IU/mL in those with HBeAg-negative CHB infection. qHBsAg levels were found to be significantly lower in HBeAg-positive CHB patients, while there was no significant difference between HBeAg-negative CHB patients and HBeAg-negative chronic HBV infection. These findings may be associated with the dynamic nature of the infection and host immune responses. The low qHBsAg levels observed in HBeAg-positive patients may be explained by mechanisms such as enhanced immune response or viral integration into the host genome [17, 18].

In our study, the diagnostic accuracy of qHBsAg levels for identifying liver fibrosis was found to be weak (ROC analysis: AUC 0.511). The cut-off value of 1024 IU/mL demonstrated high sensitivity (95.2%) but low specificity (15.1%), indicating that qHBsAg alone is insufficient for diagnosing fibrosis. These findings suggest that qHBsAg levels may be partially related to histological activity in HBeAg-positive patients, whereas this relationship appears weaker in HBeAg-negative individuals. In contrast, HBV DNA levels may have a limited role in fibrosis staging among HBeAg-negative patients.

These results highlight the need for reliable noninvasive fibrosis markers and suggest that qHBsAg should only be used in conjunction with other indicators. In a study by Gan et al. [19], qHBsAg levels were shown to contribute to fibrosis prediction; however, our findings indicate that qHBsAg alone is not a reliable predictor of histological stage. This may reflect the influence of multiple variables on the course of HBV infection, including host immune responses, genotype variations, and viral integration processes in the liver. Differences in patient population, genotype distribution, or methodology may also have contributed to these findings. Therefore, we believe that more comprehensive, prospective cohort studies including genotype data and long-term follow-up are needed.

Our results showing the limited diagnostic accuracy of qHBsAg for advanced fibrosis are in agreement with previous studies, including the cohort by Loureiro et al. [20], which demonstrated that even in patients with qHBsAg levels above 1000 IU/mL, significant fibrosis could not be reliably distinguished. These findings emphasize that qHBsAg, although reflecting viral replicative activity to some extent, may be insufficient as a stand-alone marker for fibrosis assessment.

In addition, a recent study by Yıldız et al. [21] investigated the role of qHBsAg in distinguishing chronic infection from chronic hepatitis in HBeAg-negative patients with intermediate HBV DNA levels (2000–20000 IU/mL). Their findings suggested that a qHBsAg threshold of ≤1000 IU/mL could be valid even in this subgroup and proposed that patients with qHBsAg > 20,000 IU/mL and HBV DNA > 2000 IU/mL might be considered for treatment without liver biopsy. Although our cohort had higher average qHBsAg levels, these findings support the concept that qHBsAg may help stratify patients for treatment decisions, albeit with significant limitations as demonstrated by our ROC analysis (AUC 0.511).

## 5. Conclusion

This study provides important findings by comparing the laboratory and histopathological features of different clinical forms of CHB. In particular, the limited role of qHBsAg in diagnosing fibrosis and the complex nature of viral dynamics in HBeAg-positive patients are issues that warrant further investigation. The results emphasize the importance of personalized treatment strategies in the management of CHB.

## 6. Limitations

The limitations of our study include its cross-sectional design, which prevented long-term follow-up of the cases, the absence of serial HBV DNA and HBsAg measurements, and the lack of HBV genotype analysis. Considering the dynamic nature of HBV infection, long-term monitoring of HBsAg and HBV DNA levels in conjunction with genotyping could yield more accurate conclusions. Additionally, approximately 33% of patients declined liver biopsy, which limited the ability to conduct the study with a larger dataset.

## Figures and Tables

**Table 1:** Comparison of HBeAg-negative chronic HBV infection and chronic hepatitis B (CHB) patients.

**Parameters**	**HBeAg-negative chronic HBV infection (** **n** = 142**)**	**CHB patients (** **n** = 165**)**	**p** ** value**
Age (years)	42.95 ± 13.57	41.04 ± 13.78	> 0.05
AST (U/L)	27.05 ± 18.35	59.40 ± 232.21	> 0.05
ALT (U/L)	29.97 ± 22.40	74.17 ± 223.48	0.014
ALP (U/L)	75.24 ± 38.10	74.75 ± 29.57	> 0.05
GGT (U/L)	33.03 ± 26.91	40.03 ± 77.81	> 0.05
Urea (mg/dL)	29.47 ± 7.06	30.28 ± 15.91	> 0.05
Creatinine (mg/dL)	0.66 ± 0.17	0.73 ± 0.51	> 0.05
Total protein (g/dL)	7.28 ± 0.54	7.37 ± 0.51	> 0.05
Albumin (g/dL)	4.40 ± 0.38	4.37 ± 0.45	> 0.05
Total bilirubin (mg/dL)	0.73 ± 0.33	0.80 ± 0.88	> 0.05
Direct bilirubin (mg/dL)	0.24 ± 0.13	0.27 ± 0.52	> 0.05
qHBsAg (IU/mL)	3573.58 ± 3540.83	3556.08 ± 1321.53	> 0.05
HBV DNA (IU/mL)	2767.74 ± 2941.91	2.53 × 10^8^ ± 1.4 × 10^9^	0.025

**Table 2:** Comparison of HBeAg-positive and HBeAg-negative CHB patients.

**Parameters**	**HBeAg (+) CHB (** **n** = 39**)**	**HBeAg (−) CHB (** **n** = 126**)**	**p** ** value**
Age (years)	31.56 ± 11.34	43.55 ± 13.15	< 0.001
AST (U/L)	119.76 ± 456.62	40.49 ± 54.85	0.02
ALT (U/L)	142.17 ± 409.49	53.31 ± 101.25	0.007
ALP (U/L)	96.00 ± 101.55	73.44 ± 29.43	0.035
GGT (U/L)	38.71 ± 42.82	39.71 ± 84.13	> 0.05
Urea (mg/dL)	30.05 ± 8.89	30.22 ± 17.50	> 0.05
Creatinine (mg/dL)	0.93 ± 1.33	> 0.05	> 0.05
Total protein (g/dL)	7.49 ± 0.59	7.32 ± 0.48	> 0.05
Albumin (g/dL)	4.42 ± 0.45	4.36 ± 0.45	> 0.05
Total bilirubin (mg/dL)	1.06 ± 1.43	0.73 ± 0.59	0.032
Direct bilirubin (mg/dL)	0.47 ± 1.04	0.22 ± 0.11	0.003
qHBsAg (IU/mL)	1805.17 ± 920.29	4028.61 ± 961.21	< 0.001
HBV DNA (IU/mL)	1.15 × 10^9^ ± 2.81 × 10^9^	4.9 × 10^7^ ± 4.512 × 10^8^	< 0.001

**Table 3:** Comparison of HBeAg-negative chronic HBV infection and HBeAg-positive CHB patients.

**Parameters**	**HBeAg-negative chronic HBV infection (** **n** = 142**)**	**HBeAg (+) CHB (** **n** = 39**)**	**p** ** value**
Age (years)	42.95 ± 13.57	31.56 ± 11.34	< 0.001
ALT (U/L)	29.97 ± 22.40	142.17 ± 409.49	<0.001
Total bilirubin (mg/dL)	0.73 ± 0.33	1.06 ± 1.43	0.029
Direct bilirubin (mg/dL)	0.24 ± 0.13	0.47 ± 1.04	0.004
qHBsAg (IU/mL)	3573.58 ± 3540.83	1805.17 ± 920.29	< 0.001
HBV DNA (IU/mL)	2767.74 ± 2941.91	1.15 × 10^9^ ± 2.81 × 10^9^	< 0.001

**Table 4:** Comparison of HBeAg-negative chronic HBV infection and HBeAg-negative CHB patients.

**Parameters**	**HBeAg-negative chronic HBV infection (** **n** = 142**)**	**HBeAg (−) CHB (** **n** = 126**)**	**p** ** value**
Age (years)	42.95 ± 13.57	43.55 ± 13.15	> 0.05
ALT (U/L)	29.97 ± 22.40	53.31 ± 101.25	> 0.05
HBV DNA (IU/mL)	2767.74 ± 2941.91	4.9 × 10^7^ ± 4.512 × 10^8^	> 0.05
qHBsAg (IU/mL)	3573.58 ± 3540.83	4028.61 ± 961.21	> 0.05

**Table 5:** Comparison of qHBsAg and HBV DNA levels by histological scores in HBeAg-positive and HBeAg-negative patients.

**Group**		**HAI-1 (** **n** **)**	**HAI-2 (** **n** **)**	**p**	**ISHAK-1 (** **n** **)**	**ISHAK-2 (** **n** **)**	**p**
HBeAg (+)	QHBsAg (IU/mL)	1375 ± 564 (12)	2103 ± 851 (19)	0.008	1629 ± 781 (20)	2172 ± 822 (11)	0.079
	HBV DNA (IU/mL)	1.86 × 10^9^ ± 3.97 × 10^9^	3.50 × 10^8^ ± 5.33 × 10^8^	0.109	1.24 × 10^9^ ± 3.15 × 10^8^	3.73 × 10^8^ ± 4.34 × 10^7^	0.237
HBeAg (−)	QHBsAg (IU/mL)	3990 ± 1073 (39)	3883 ± 869 (41)	0.628	3971 ± 1044 (57)	3863 ± 781 (22)	0.622
	HBV DNA (IU/mL)	5.10 × 10^6^ ± 1.87 × 10^7^	1.73 × 10^7^ ± 6.73 × 10^7^	0.268	1.51 × 10^6^ ± 5.36 × 10^5^	3.58 × 10^7^ ± 9.17 × 10^6^	0.006

## Data Availability

Due to privacy/ethical concerns, supporting data cannot be made openly available. However, anonymized data may be shared by the corresponding author upon justified request.
